# Bilateral, Dissimilar, Impacted Supernumerary Teeth in a Non-syndromic Child: A Case Report

**DOI:** 10.7759/cureus.89109

**Published:** 2025-07-31

**Authors:** Majd Morad, Marwa Alhothali, Yasser Ibrahim, Afnan S Aljohani

**Affiliations:** 1 Dentistry, Umm Al-Qura University, Makkah, SAU; 2 Pediatric Dentistry, Security Forces Hospital, Makkah, Mecca, SAU; 3 Maxillofacial Surgery, Security Forces Hospital, Makkah, Mecca, SAU

**Keywords:** dental anomalies, impacted teeth, non-syndromic, pediatric patient, supernumerary teeth, surgical intervention

## Abstract

Supernumerary teeth are additional teeth that develop beyond the normal dentition and can appear in various forms and locations. While often asymptomatic, impacted supernumerary teeth may interfere with the eruption of permanent teeth or displace adjacent structures. This case report presents a rare occurrence of bilateral, morphologically dissimilar, impacted supernumerary teeth in a non-syndromic pediatric patient. Following comprehensive clinical and radiographic evaluations, a definitive diagnosis was made. Surgical removal of the impacted teeth was performed under general anesthesia, followed by scheduled follow-up visits to evaluate treatment outcomes, monitor the case progress, and ensure proper eruption of the permanent teeth. This case underscores the importance of early diagnosis, individualized treatment planning, and regular follow-up. Although based on a single case, the favorable outcome supports the potential benefits of timely intervention in similar clinical scenarios.

## Introduction

Odontogenesis begins between the sixth and eighth weeks of intrauterine life and progresses through multiple stages, including initiation, proliferation, histodifferentiation, morphodifferentiation, apposition, and calcification [1,2]. Dental anomalies arise when disruptions occur during these stages, particularly during initiation and proliferation. Supernumerary teeth, which are extra teeth beyond the normal dentition, are one such anomaly. Although their exact cause remains unclear, localized hyperactivity of the dental lamina is the most widely accepted theory [1,2].

Hyperdontia affects individuals of all races, with an incidence of 0.3%-0.8% in primary dentition and 0.1%-3.8% in permanent dentition, and is approximately twice as common in males [3,4]. A Saudi Arabian study reported a 0.5% prevalence in children aged 6-18 years, with most cases involving anterior maxillary teeth [5].

Supernumerary teeth are classified by shape and location. Supplemental types resemble normal teeth, while rudimentary forms include conical, tuberculate, molariform, and odontoma-like shapes [6]. Approximately 90% occur in the maxilla, particularly in the anterior region (mesiodens), followed by the molar area (paramolars) [4,6]. Conical mesiodens are the most common, accounting for about 75% of cases [7]. Tuberculate types, comprising roughly 12%, typically appear palatally in pairs and may have incomplete roots. Bilateral multiple supernumerary teeth in the premaxillary region are less common than single occurrences and rarely erupt [7,8].

Supernumerary teeth represent a relatively frequent developmental anomaly in pediatric dentistry and may lead to a variety of complications if not identified early. Their detection, classification, and management are key components of pediatric oral healthcare. According to the American Academy of Pediatric Dentistry, timely evaluation and appropriate surgical intervention are essential in minimizing the risks associated with these anomalies, especially when they are impacted or causing functional or esthetic concerns [9]. Recent literature emphasizes that early diagnosis, aided by radiographic techniques, plays a crucial role in preventing malocclusion and eruption disturbances caused by supernumerary teeth [10].

This report describes a rare case of non-syndromic, bilateral impacted supernumerary teeth in the premaxillary region, one conical and one tuberculate, and emphasizes the clinical implications and rationale for early surgical management.

## Case presentation

**Figure 1:**
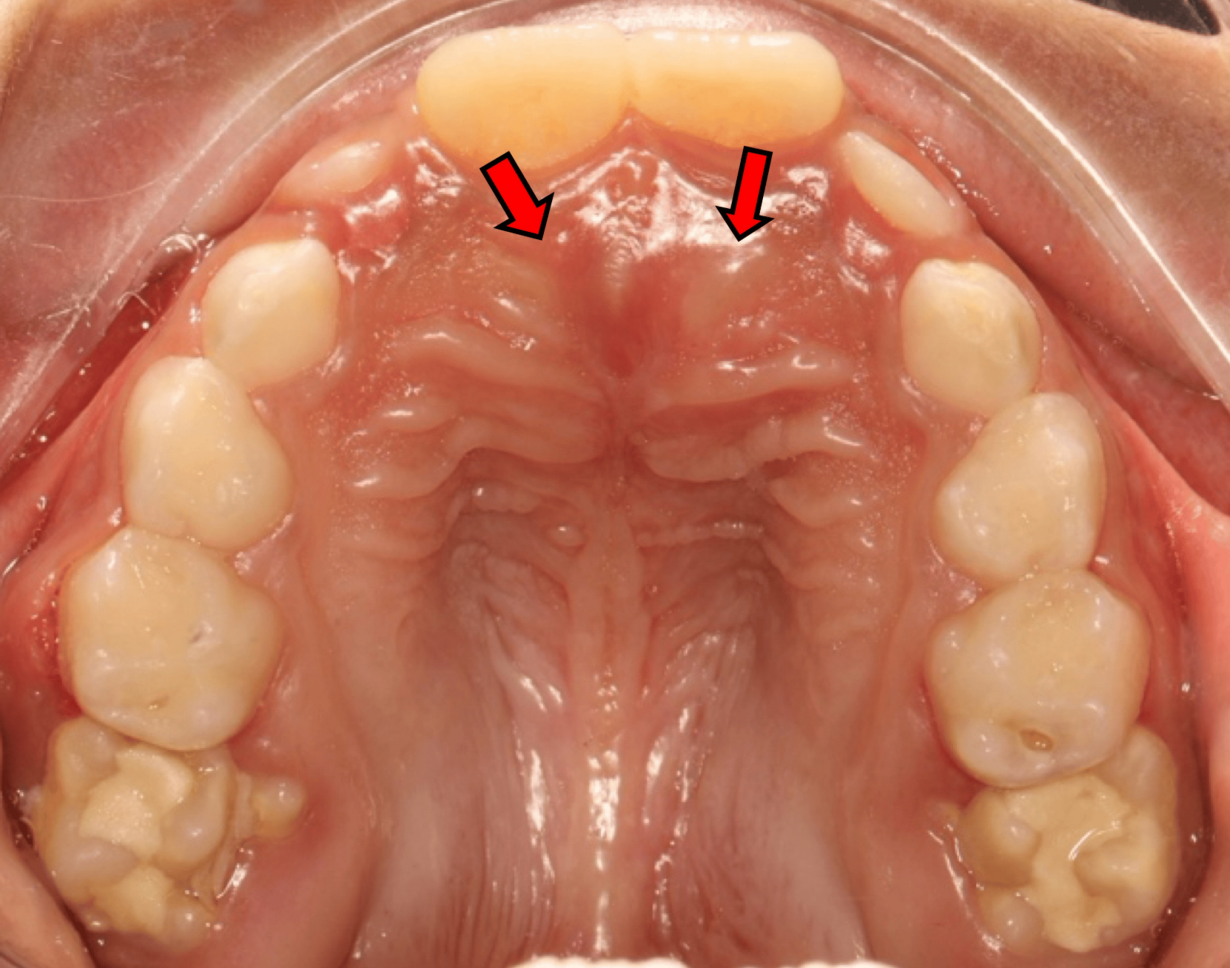
Intraoral occlusal photograph The red arrows indicate the palatal bulges observed in the premaxillary region, corresponding to the suspected location of unerupted supernumerary teeth.

A seven-year-old male patient presented to the pediatric dental clinic at the Security Forces Hospital (SFH) in Makkah, Saudi Arabia, accompanied by his mother. She reported concerns about visible dental decay in her child’s primary molars. The patient’s medical history was significant for asthma, which was well-managed and not currently affecting his overall health. During the intraoral examination, multiple carious lesions were noted affecting both the primary and developing permanent molars. A dental abscess was associated with the upper right second primary molar. Additionally, a mild swelling was detected in the premaxillary area. On palpation, a firm, immobile mass was felt beneath the mucosa, raising suspicion of unerupted supernumerary teeth (Figure 1).

The patient’s oral hygiene was assessed as poor, and he exhibited a tongue-thrusting habit, which was also noted during the clinical assessment.

Investigation and diagnosis

**Figure 2:**
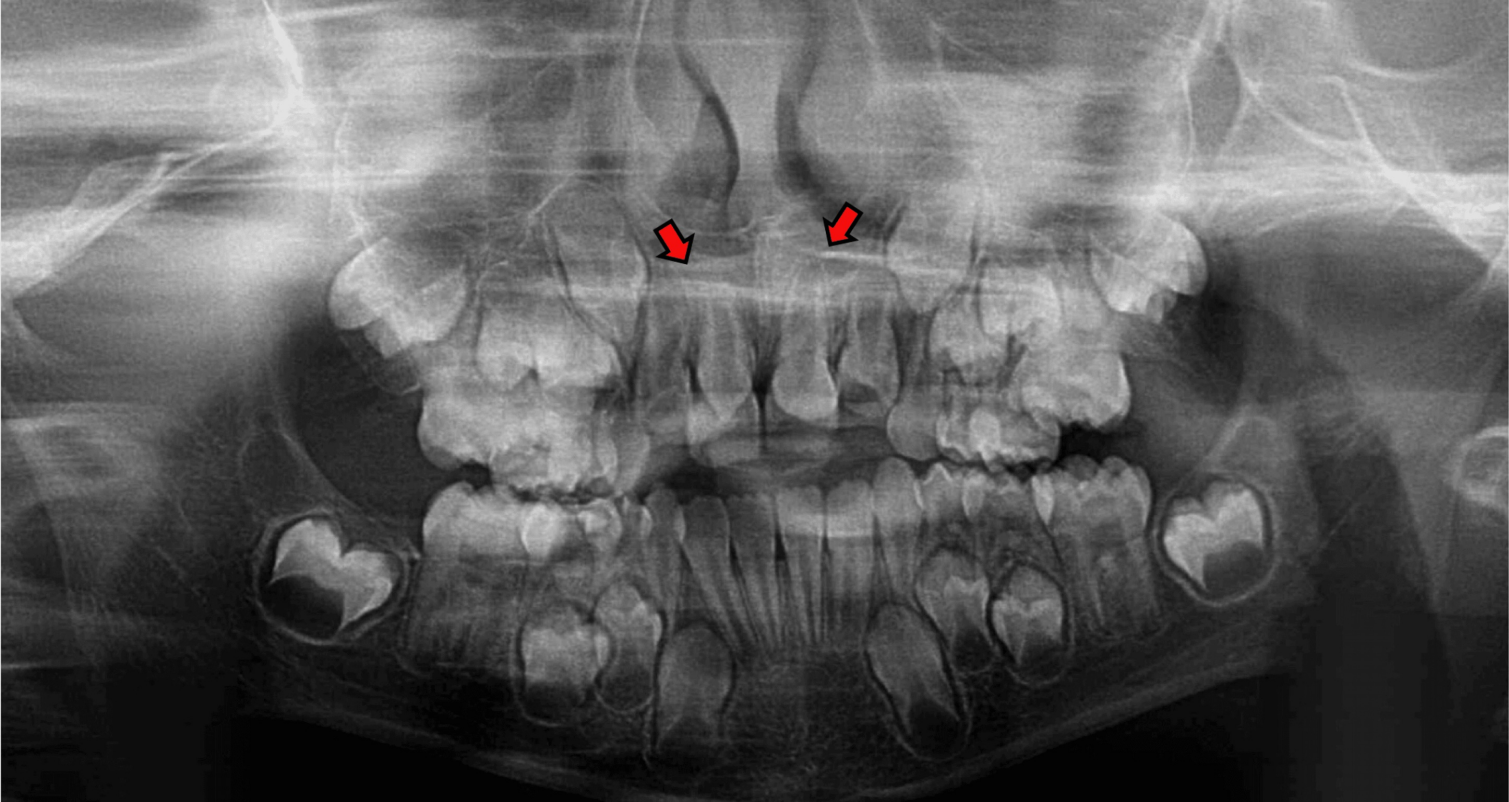
Panoramic radiograph The red arrows highlight the presence of two impacted supernumerary teeth located in the anterior maxillary region, positioned palatally to the permanent central incisors.

A routine panoramic radiograph was obtained, revealing two impacted supernumerary teeth situated in the maxillary anterior region (Figure 2).

**Figure 3:**
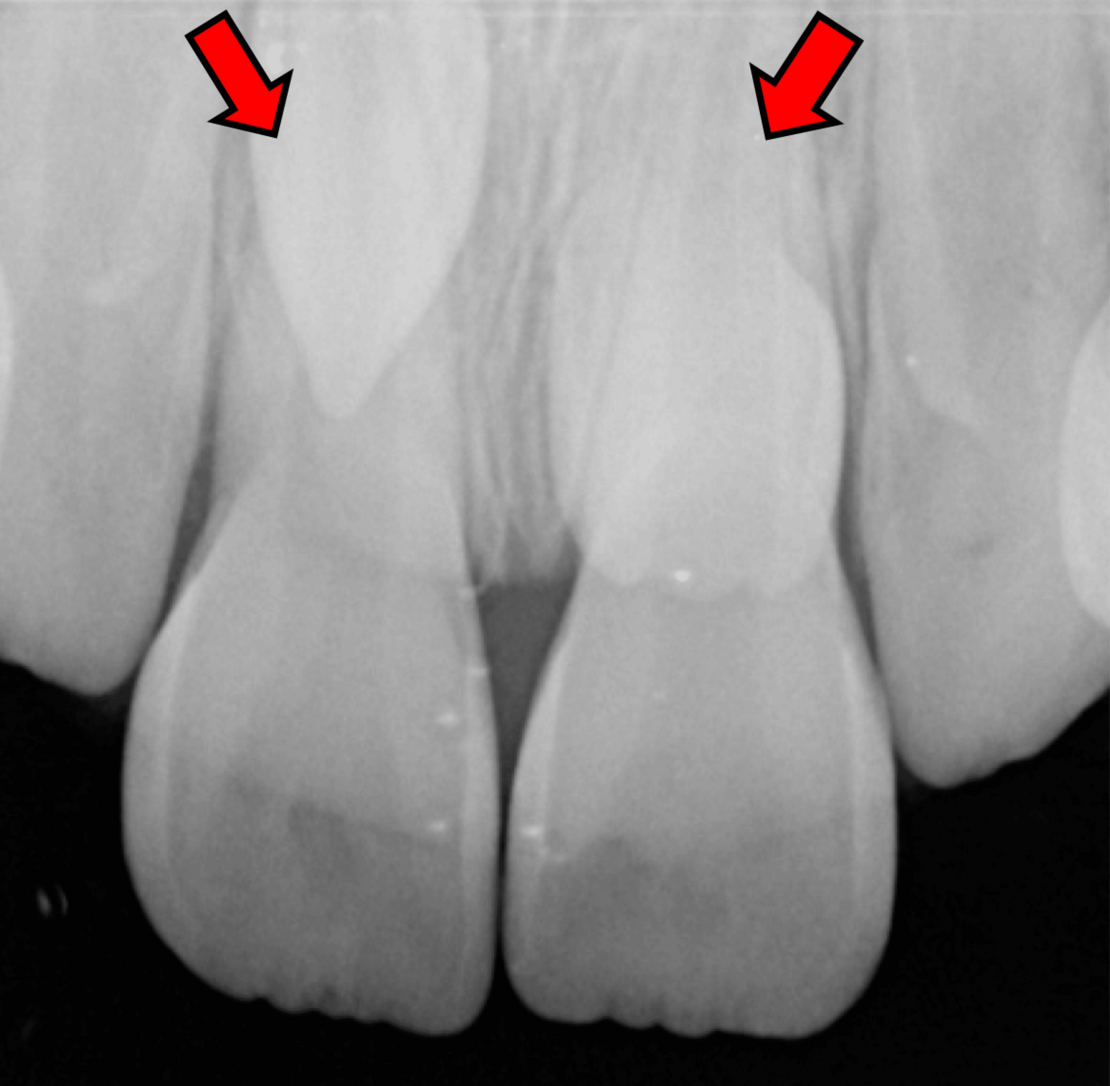
Periapical radiograph The red arrows point to the impacted supernumerary teeth, revealing their orientation and proximity to the roots of the permanent maxillary central incisors.

This initial finding prompted further imaging, including a periapical radiograph to better localize the teeth and assess their relationship with the surrounding structures (Figure 3).

**Figure 4:**
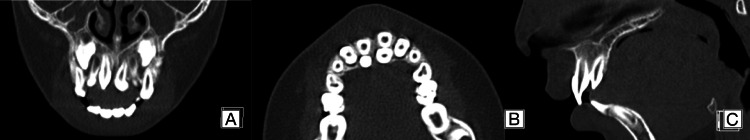
CT scans of impacted supernumerary teeth in the maxilla (A) Coronal view showing the distinct morphology of the impacted supernumerary teeth: conical on the right and tuberculate on the left. (B) Axial view revealing the horizontal position of the supernumerary teeth palatal to the central incisors. (C) Sagittal view illustrating the close proximity of the supernumerary teeth to the roots of the maxillary central incisors, with evidence of root displacement.

To gain a more comprehensive view, a computed tomography (CT) scan was performed. The coronal CT section confirmed the presence of two distinct impacted supernumerary teeth, one with a conical morphology on the right side and another with a tuberculate shape on the left (Figure 4A). Sagittal and axial CT images revealed an abnormal spatial relationship between these supernumerary teeth and the permanent central incisors, including displacement of the incisor roots and loss of facial bone support (Figures 4B, 4C).

Additionally, the images indicated incomplete root development in the permanent incisors compared to the fully formed roots of the supernumerary teeth. The comprehensive clinical and radiographic examinations indicated the need for extensive dental intervention. A multidisciplinary team, including specialists in pediatric dentistry, orthodontics, and oral and maxillofacial surgery, recommended the immediate surgical removal of the impacted supernumerary teeth. This decision was based on the risk of complications such as labial displacement, root resorption, and potential concrescence involving the maxillary central incisors. A detailed treatment plan was formulated, and the necessary consent forms were prepared after thoroughly reviewing the patient’s medical and dental history. The proposed plan and associated procedures were carefully discussed with the patient’s mother, who agreed to proceed with treatment.

Treatment

**Figure 5:**
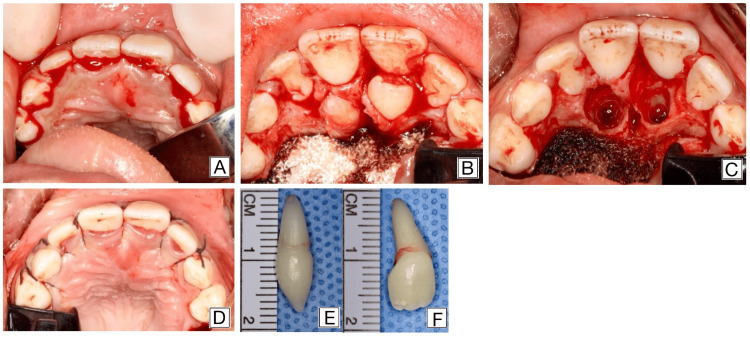
Surgical removal of the supernumerary teeth. (A) Sulcular incision and flap elevation in the maxillary anterior region. (B) Exposure of the impacted supernumerary teeth. (C) Surgical site after removal of the teeth. (D) Postoperative view showing flap repositioning and sutures. (E) Extracted conical-shaped supernumerary tooth. (F) Extracted tuberculate-shaped supernumerary tooth with enlarged crown and root morphology.

The treatment commenced with full-mouth dental rehabilitation, addressing the patient’s carious lesions and abscess. Once stabilized, the surgical phase began with a conservative ‏full thickness palatal mucoperiosteal flap. A sulcular incision from the first molar on one side to the other was made in the palatal mucosa to access the impacted area (Figure 5A), and a mucoperiosteal flap was carefully elevated to expose the underlying teeth (Figure 5B). Both supernumerary teeth were removed using a conservative extraction technique without the need for bone removal, thus preserving the integrity of the alveolar bone and avoiding root exposure of the adjacent permanent teeth (Figure 5C). The surgical site was sutured using resorbable 3-0 sutures to ensure optimal healing (Figure 5D). The extracted teeth were then examined: one showed a conical shape, while the other exhibited an abnormal crown and root form with increased buccopalatal width, consistent with a tuberculate supernumerary tooth (Figure 5F).

Postoperative care included instructions for a soft, cold diet, antiseptic mouth rinse, and good oral hygiene practices.

Follow-up

**Figure 6:**
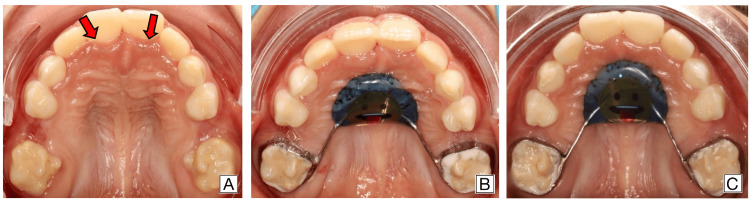
Postoperative intraoral photographs. (A) Two weeks post-surgery. Red arrows indicate the healed area where the palatal bulge was previously observed. (B) One-month follow-up showing the placement of an upper Nance appliance. (C) Six-month follow-up confirming proper healing and maintenance of space for future eruption.

Comprehensive oral rehabilitation was carried out, including caries control and surgical intervention, followed by the placement of an upper Nance appliance to maintain the space of the extracted upper primary second molar. The patient returned for suture removal and clinical evaluation two weeks postoperatively, with uneventful healing and no signs of complications. Follow-up visits at one and six months confirmed favorable outcomes, with stable tissue healing and progressive eruption of the permanent incisors, documented through clinical photographs at each stage (Figure 6).

## Discussion

The etiology of supernumerary teeth remains uncertain, with several theories proposed, including atavism, dental lamina hyperactivity, dichotomy of the tooth bud, and the progress zone concept. Among these, localized hyperactivity of the dental lamina is the most widely accepted explanation [11,12]. Genetic factors are also implicated, particularly in syndromic cases such as Gardner syndrome, cleidocranial dysplasia, and cleft lip/palate. However, bilateral supernumerary teeth in non-syndromic patients, as observed in this case, are considered rare [2,7].

Supernumerary teeth are frequently asymptomatic and often discovered incidentally during routine radiographic evaluations. Early diagnosis typically leads to better outcomes by preventing complications such as delayed eruption, malalignment, root resorption, and cyst formation [11-13]. Imaging modalities like computed tomography (CT) and cone beam CT (CBCT) are essential for accurate localization and assessment, especially in complex or impacted cases [14].

In this report, the patient presented with two impacted supernumerary teeth: a conical-shaped mesiodens on the right and a tuberculate tooth on the left. Tuberculate supernumerary teeth are more commonly associated with complications, rarely erupt, and often appear bilaterally, accounting for approximately 12% of cases [7,10]. In contrast, conical types, seen in about 75%, are more likely to erupt spontaneously.

The coexistence of two distinct types of supernumerary teeth in a non-syndromic pediatric patient is uncommon. Most cases involve a single supernumerary tooth (76%-86%), while double or multiple occurrences are less frequent, particularly in non-syndromic individuals [8,9]. When these teeth are located palatally near the central incisors, they can interfere with eruption and alignment. Although the patient’s permanent incisors showed no eruption delay, CT scans revealed anterior displacement of the roots, creating a risk of periodontal compromise and potential tooth loss. Only about 25% of mesiodens erupt spontaneously, and surgical removal is typically required when they are impacted [9].

The treatment options and the timing of treatment for supernumerary teeth depend on several factors, including their location, number, morphology, and potential impact on adjacent teeth and eruption patterns. In asymptomatic cases where the supernumerary teeth do not interfere with the eruption of permanent teeth, careful monitoring may be sufficient. However, early intervention is often recommended when there is a risk of complications [15,16]. The importance of timely decision-making lies in the fact that early surgical removal, ideally during the mixed dentition phase (ages 6-8), can facilitate the spontaneous eruption of permanent teeth and prevent long-term complications. However, in certain situations, delayed intervention may be preferred to allow for full root development of adjacent teeth, thereby reducing the risk of iatrogenic damage [16,17]. Not all cases, however, warrant immediate removal; asymptomatic and non-interfering supernumerary teeth may be monitored periodically, especially when they pose no threat to the eruption path or occlusion of the permanent dentition [18]. Nonetheless, such conservative approaches demand careful follow-up and clearly defined criteria for future surgical intervention.

In terms of treatment methods, the surgical approach for impacted supernumerary teeth using a full-thickness mucoperiosteal flap provides effective exposure and minimizes postoperative complications [16]. Such techniques should be considered in cases involving deeply positioned or palatally impacted teeth, as in the current case.

The American Academy of Pediatric Dentistry emphasizes individualized treatment planning [9]. In this case, the decision to proceed with early surgical intervention was supported by both clinical and radiographic findings, aiming to prevent long-term complications and facilitate the proper alignment of permanent incisors.

Nevertheless, concerns persist about disrupting root development and inducing dental anxiety in young children. Parents may hesitate to approve surgery if the condition appears asymptomatic. However, delaying treatment increases the risk of complications such as malocclusion, root resorption, or the need for complex orthodontic treatment. Here, the role of the pediatric dentist becomes essential not only in clinical diagnosis and treatment planning but also in educating parents about the potential consequences of interaction. Clear communication and counseling can help caregivers make informed decisions that prioritize the child’s long-term oral health [7,17,18].

## Conclusions

Early detection and individualized management of supernumerary teeth are crucial to preventing long-term complications. In the presented case, the early identification of two impacted supernumerary teeth allowed for timely conservative surgical removal under general anesthesia, which facilitated proper alignment of the adjacent permanent teeth and minimized the risk of root resorption. This case underscores the importance of thorough clinical and radiographic assessment, as well as clear communication with parents. While observation may be appropriate in some asymptomatic cases, early intervention remains the preferred approach when there is a risk of eruption disturbance or adjacent tooth damage.

## References

[1] Vargas Vargas, Alexia Maylid, Mendez Mendez (2024). Supernumerary teeth in pediatric patients: an updated scoping review. Int J Appl Dent Sci.

[2] Meade MJ (2020). Supernumerary teeth: an overview for the general dental practitioner. Dent Update.

[3] He L, Que G, Yang X, Yan S, Luo S (2023). Prevalence, clinical characteristics, and 3-dimensional radiographic analysis of supernumerary teeth in Guangzhou, China: a retrospective study. BMC Oral Health.

[4] Sholapurmath SM, Bharuka SB, Jasani B (2014). Unerupted maxillary anterior supernumerary tooth - a surgical intervention. Indian J Dent Adv.

[5] Bakhurji EA, Aldossary F, Aljarbo J, AlMuhammadi F, Alghamdi M, Nazir MA (2021). Prevalence and distribution of nonsyndromic dental anomalies in children in eastern Saudi Arabia: a radiographic study. ScientificWorldJournal.

[6] Davidson CL, Smit C, Nel S (2025). Supernumerary teeth: a pictorial review and revised classification. J Oral Biol Craniofac Res.

[7] Shah A, Gill DS, Tredwin C, Naini FB (2008). Diagnosis and management of supernumerary teeth. Dent Update.

[8] Eigbobo JO, Osagbemiro BB (2011). Bilateral tuberculate supernumerary teeth. Clin Pract.

[9] American Academy of Pediatric Dentistry (2018). Management considerations for pediatric oral surgery and oral pathology. Pediatr Dent.

[10] Mallineni SK (2014). Supernumerary teeth: review of the literature with recent updates. Conf Pap Sci.

[11] Arandi NZ (2020). Hyperdontia: exploring the developmental abnormality. J Pre Clin Clin Res.

[12] Jham BC, Costa NL, Batista AC, Mendonça EF (2014). Traumatic neuroma of the mandible: a case report with spontaneous remission. J Clin Exp Dent.

[13] Dias GF, Hagedorn H, Latta Maffezzolli MD, de Freitas da Silva F, Teixeira Alves FB (2019). Diagnosis and treatment of supernumerary teeth in the pediatric clinic: case report. Rev Cefac.

[14] Mallineni SK, Anthonappa RP, Jayaraman J, King NM (2025). Radiographic localization of supernumerary teeth: a narrative review. Front Dent Med.

[15] Acharya S (2015). Supernumerary teeth in maxillary anterior region: report of three cases and their management. Int J Sci Study.

[16] Alsweed AA, Al-Sughier Z (2020). Surgical management of unerupted permanent maxillary central incisors due to presence of two supernumerary teeth. Int J Clin Pediatr Dent.

[17] Gupta S, Marwah N (2012). Impacted supernumerary teeth-early or delayed intervention: decision making dilemma?. Int J Clin Pediatr Dent.

[18] Scheiner MA, Sampson WJ (1997). Supernumerary teeth: a review of the literature and four case reports. Aust Dent J.

